# Beta-blockers and renin-angiotensin system inhibitors in acute myocardial infarction managed with inhospital coronary revascularization

**DOI:** 10.1038/s41598-020-72232-y

**Published:** 2020-09-16

**Authors:** Hui Wen Sim, Huili Zheng, A. Mark Richards, Ruth W. Chen, Anders Sahlen, Khung-Keong Yeo, Jack W. Tan, Terrance Chua, Huay Cheem Tan, Tiong Cheng Yeo, Hee Hwa Ho, Boon-Wah Liew, Ling Li Foo, Chi-Hang Lee, Derek J. Hausenloy, Mark Y. Chan

**Affiliations:** 1 Department of Cardiology, National University Heart Centre Singapore, 1E Kent Ridge Road, NUHS Tower Block, Level 9, Singapore, 119228 Singapore; 2 Department of Medicine, Ng Teng Fong General Hospital, 1 Jurong East Street 21, Singapore, 609606 Singapore; 3 Health Promotion Board, National Registry of Disease Office, 3 Second Hospital Ave, Singapore, 168937 Singapore; 4 Yong Loo Lin School of Medicine, National University of Singapore, 10 Medical Dr, Singapore, 117597 Singapore; 5 Cardiovascular Research Institute, 1E Kent Ridge Road, Singapore, 119228 Singapore; 6 Department of Cardiology, Tan Tock Seng Hospital, 11 Jln Tan Tock Seng, Singapore, 308433 Singapore; 7 National Heart Centre Singapore, 5 Hospital Dr, Singapore, 169609 Singapore; 8 Karolinska Institutet, Stockholm, Sweden; 9 Department of Cardiology, Changi General Hospital, 2 Simei Street 3, Singapore, 529889 Singapore; 10 Cardiovascular and Metabolic Disorders Program, Duke-National University of Singapore Medical School, Singapore, Singapore; 11 National Heart Research Institute Singapore, National Heart Centre, Singapore, Singapore; 12 The Hatter Cardiovascular Institute, University College London, London, UK; 13 Cardiovascular Research Center, College of Medical and Health Sciences, Asia University, Taichung City, Taiwan

**Keywords:** Cardiology, Outcomes research

## Abstract

Pivotal trials of beta-blockers (BB) and angiotensin converting enzyme inhibitors/angiotensin receptor blockers (ACEI/ARB) in acute myocardial infarction (AMI) were largely conducted prior to the widespread adoption of early revascularization. A total of 15,073 patients with AMI who underwent inhospital coronary revascularization from January 2007 to December 2013 were analyzed. At 12 months, BB was significantly associated with a lower incidence of major adverse cardiovascular events (MACE, adjusted HR 0.80, 95% CI 0.70–0.93) and all-cause mortality (adjusted HR 0.69, 95% CI 0.55–0.88), while ACEI/ARB was significantly associated with lower all-cause mortality (adjusted HR 0.80, 95% CI 0.66–0.98) and heart failure (HF) hospitalization (adjusted HR 0.80, 95% CI 0.68–0.95). Combined BB and ACEI/ARB use was associated with the lowest incidence of MACE (adjusted HR 0.70, 95% CI 0.57–0.86), all-cause mortality (adjusted HR 0.55, 95% CI 0.40–0.77) and HF hospitalization (adjusted HR 0.64, 95% CI 0.48–0.86). This were consistent for left ventricular ejection fraction < 50% or ≥ 50%. In conclusion, in AMI managed with revascularization, both BB and ACEI/ARB were associated with a lower incidence of 12-month all-cause mortality. Combined BB and ACEI/ARB was associated with the lowest incidence of all-cause mortality and HF hospitalization.

## Introduction

Beta-blockers (BB) and angiotensin-converting enzyme inhibitors/angiotensin receptor blockers (ACEI/ARB) have been the cornerstone of treatment for acute myocardial infarction (AMI) due to their salutary effects on short- and long-term mortality. However, the majority of clinical trials showing benefit with BB and ACEI/ARB in AMI were performed before the widespread adoption of primary percutaneous coronary intervention (PCI) for ST-segment elevation MI (STEMI) and early invasive management for non-STEMI^1–4^. Even with more contemporary clinical trials of ACEI/ARB, many patients may not have undergone early revascularization^5–7^.

Contemporary observational data suggest an absence of mortality benefit from BB among patients with AMI and preserved left ventricular ejection fraction (LVEF)^8–12^. A meta-analysis found that BB reduced mortality in the pre-reperfusion era but not in reperfusion era^13^. As a consequence, the European Society of Cardiology has downgraded its recommendation of routine BB administration for AMI from class I to class IIa^14–16^. In contrast, ACEI/ARB have been shown to reduce mortality in AMI but a class I recommendation is only assigned to patients with anterior myocardial infarction (MI), heart failure (HF) and/or reduced LVEF^3–6,14–16^. In patients with recent AMI, chronic upregulation of neurohormonal activity in the sympathetic nervous system and renin–angiotensin–aldosterone system exerts deleterious effect on the myocardium, leading to pump failure. BB and ACEI/ARB downregulate sympathetic and renin-angiotensin system overactivity to improve left ventricular function and long-term clinical outcome^17,18^.

As such, there is a need to reassess the role of BB and ACEI/ARB in this contemporary treatment era of routine early revascularization for AMI. Therefore, we evaluated the association of BB and ACEI/ARB with 12-month mortality, HF hospitalization and MI in patients with AMI who had undergone inhospital coronary revascularization.

## Methods

### Data sources and study population

Patients diagnosed with STEMI or non-STEMI during their index admission from January 2007 to December 2013 were identified from the Singapore Myocardial Infarct Registry (SMIR, https://www.nrdo.gov.sg/publications/ami). AMI cases (*International Classification of Diseases Ninth Revision* and *Tenth Revision)* were identified from hospital discharge records, troponin test results, reimbursement claims and the national death registry by trained coordinators from the SMIR. Healthcare legislature in Singapore mandates that all patients diagnosed with AMI are enrolled in the SMIR with the exception of patients who opt out of enrolment. This study complies to the Helsinki declaration and was approved by the National Healthcare Group Domain Specific Review Board which allowed for a waiver of written informed consent on condition that all analyses were performed onsite at the SMIR using de-identified data.

We included all patients with a primary diagnosis of AMI and who received inhospital coronary revascularization by PCI or coronary artery bypass graft surgery (CABG) during the index hospitalization. We excluded (1) patients who were admitted for non-AMI condition but had AMI during hospitalization, (2) AMI that were not clearly classified (not STEMI or non-STEMI), (3) patients who did not receive inhospital revascularization, and (4) patients who died during index hospitalization.

### Data collection and clinical outcomes

Information on demographics, co-morbidities, history of coronary revascularization, clinical presentation, inpatient laboratory values, LVEF and pharmacotherapy on discharge were prospectively collected by trained coordinators according to a standardized case report form (https://www.nrdo.gov.sg/docs/default-source/Disease-Notification—AMI/nrdo-f004-09b-(smir-notification-form)web.pdf?sfvrsn=0). Prior to 2008, LVEF data in the registry was captured in binary format (LVEF < 50% vs ≥ 50%). From 2008 onwards, LVEF was captured as continuous data. The outcome of interest was major adverse cardiovascular events (MACE), which we defined as a composite of all-cause mortality, hospitalization for HF or hospitalization for MI, and the individual component endpoints. Death endpoints were ascertained through data linkage with the Ministry of Home Affairs Death Registry while MI hospitalization and HF hospitalization were ascertained by linking SMIR data with the Ministry of Health Mediclaims data. Only the first hospitalization for HF or MI after discharge was included and time to hospitalization was computed as the number of days from the discharge date of the index admission to the readmission date.

### Statistical analysis

For descriptive analyses, we compared baseline demographic and clinical characteristics of patients stratified to BB versus no BB and ACEI/ARB versus no ACEI/ARB. Categorical variables are shown using frequencies and percentages, and continuous variables are presented using median and interquartile range. Differences between the groups were compared by using Chi-square test for categorical variables and Mann–Whitney–Wilcoxon nonparametric test for continuous variables. Multivariable Cox proportional hazard regression models were constructed to estimate the hazard ratio (HR) and 95% confidence interval (CI) for the risk of composite endpoint, all-cause mortality, MI and HF hospitalization, for patients who were given (1) BB and those who were not given (reference group) and (2) ACEI/ARB compared to those who were not given these medications (reference group). Included in the multivariable models were age, gender, ethnicity, hypertension, diabetes, hyperlipidemia, history of MI/PCI/CABG, smoking status, Killip class on admission, creatinine level on admission and in-hospital LVEF < 50%. We further constructed another similar multivariable Cox proportional hazard regression model for patients who received both BB and ACEI/ARB (BB + ACEI/ARB), BB only, ACEI/ARB only, comparing them with the reference group of patients were on neither BB nor ACEI/ARB (no BB + no ACEI/ARB group). Competing risks from death was accounted for all hospitalization outcomes^19^. Secondary subgroup analysis examined clinical outcomes stratified by the following categories: types of AMI (STEMI or NSTEMI), age (< 65 years old or ≥ 65 years old), sex (male or female), history of diabetes, history of hypertension, Killip class on presentation (I/II or III/III), LVEF during hospitalization (< 50% or ≥ 50%), PCI during hospitalization and CABG during hospitalization. All tests were performed with STATA SE software, version 13. For all analyses, a two‐sided *P* < 0.05 was considered statistically significant.

## Results

### Baseline characteristics

**Figure 1:**
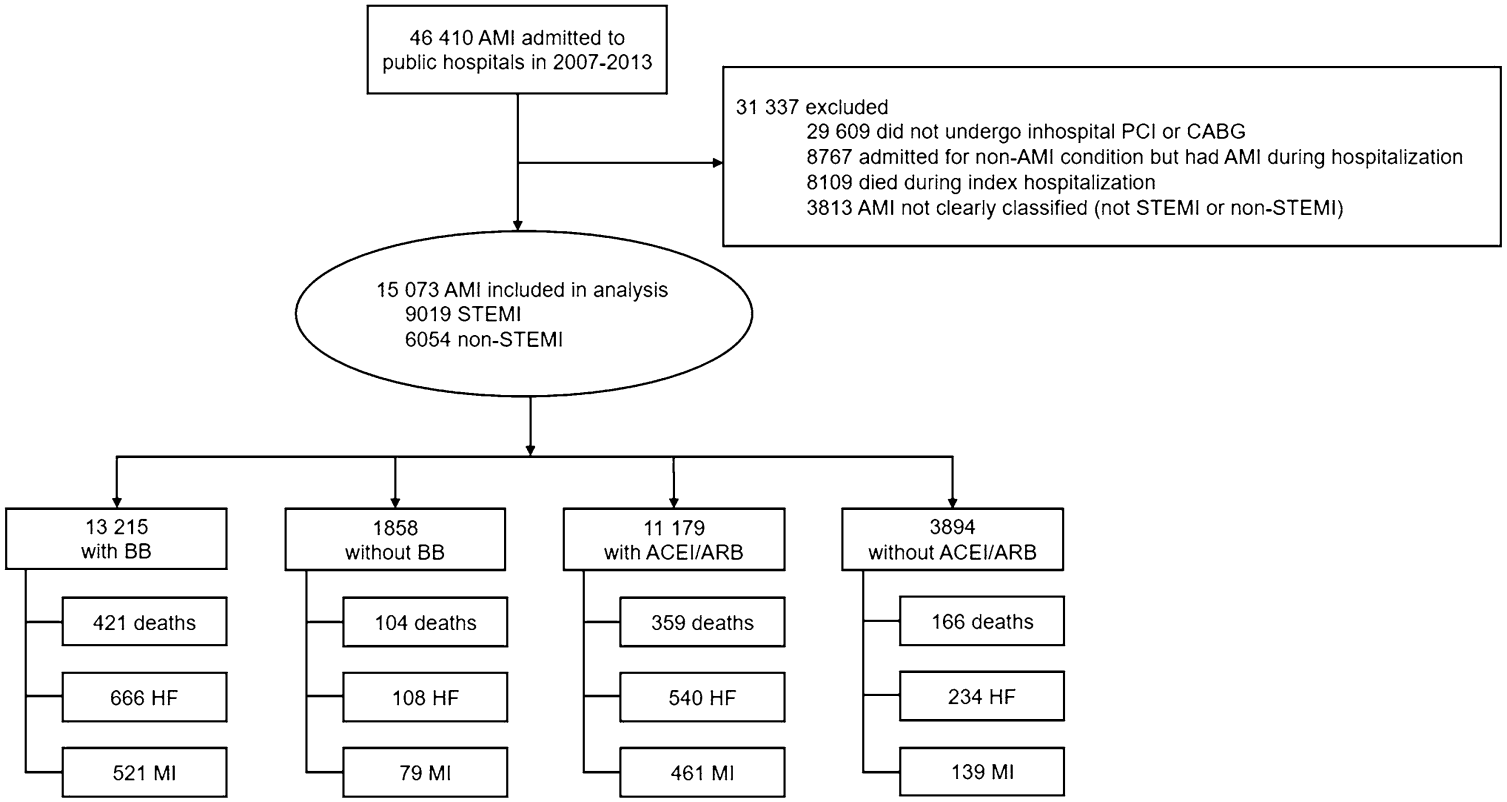
Study flow diagram. *ACEI/ARB* Angiotensin converting enzyme inhibitors/angiotensin receptor blockers, *AMI* acute myocardial infarction, *BB* beta-blockers, *CABG* coronary artery bypass graft, *CI* confidence interval, *HF* heart failure, *HR* hazard ratio, *LVEF* left ventricular ejection fraction, *MACE* major adverse cardiovascular events, *MI* myocardial infarction, *NSTEMI* non-ST-segment elevation myocardial infarction, *STEMI* ST-segment elevation myocardial infarction, *PCI* percutaneous coronary intervention.

Out of 46,410 patients with AMI from January 2007 to December 2013, 15,073 patients were included in the present analysis, of which 9,019 (59.8%) patients had STEMI and 6,054 (40.2%) of patients had non-STEMI (Fig. 1). All patients in this study cohort underwent inpatient revascularization: 14,168 (94%) had PCI while 1,024 (6.8%) had CABG. Among these patients, 119 (0.8%) had both PCI and CABG during their index hospitalization. The median time from admission to revascularization was 1 day (interquartile range 0–2 days). A total of 13,215 (87.7%) patients were on BB and 11,179 (74.2%) patients were on ACEI/ARB. Among them, 10,063 (66.8%) were on both BB and ACEI/ARB (BB + ACEI/ARB), 3,152 (20.9%) were only on BB (BB only), 1,116 (7.4%) were only on ACEI/ARB (ACEI/ARB only), and 742 (4.9%) were on neither BB nor ACEI/ARB (no BB + no ACEI/ARB group).

Table 1 illustrates the baseline characteristics of the study cohort. Those who were prescribed BB were younger, more likely to be men, have hypertension and more likely to have impaired LVEF < 50% than those who were not. Patients who were prescribed ACEI/ARB were more likely to have diabetes, hypertension, hyperlipidemia, previous MI, previous PCI, previous CABG and STEMI at presentation than those who were not.

**Table 1:** Baseline demographics, clinical presentation and medication given at discharge.

	All patients (N = 15,073)	Discharge BB			Discharge ACEI/ARB		
		Yes (n = 13,215)	No (n = 1,858)	*P* value	Yes (n = 11,179)	No (n = 3,894)	*P* value
**Baseline demographics**							
Age in years, median (IQR)	58 (51–66)	58 (51–66)	60 (52–70)	<.001	58 (51–66)	58 (51–66)	.601
Male, n (%)	12,537 (83.2)	11,036 (83.5)	1,501 (80.8)	.003	9,320 (83.4)	3,217 (82.6)	0.28
**Race**							
Chinese, n (%)	9,422 (62.5)	8,254 (62.5)	1,168 (62.9)	.908	6,955 (62.2)	2,467 (63.4)	.020
Malay, n (%)	2,901 (19.3)	2,549 (19.3)	352 (19.0)		2,130 (19.1)	771 (19.8)	
Indian, n (%)	2,499 (16.6)	2,195 (16.6)	304 (16.4)		1,914 (17.1)	585 (15.0)	
Others	251 (1.6)	217 (1.6)	34 (1.8)		180 (1.6)	71 (1.8)	
Current/former smoker, n (%)	9,274 (61.7)	8,048 (61.1)	1,226 (66.2)	< .001	6,869 (61.6)	2,405 (62.1)	0.60
History of diabetes, n (%)	4,725 (31.4)	4,162 (31.5)	563 (30.4)	.31	3,605 (32.3)	1,120 (28.8)	< .001
History of hypertension, n (%)	8,661 (57.5)	7,683 (58.2)	978 (52.8)	< .001	6,771 (60.6)	1,890 (48.6)	< .001
Hyperlipidemia, n (%)	7,884 (52.4)	6,894 (52.2)	990 (53.4)	.35	5,930 (53.1)	1,954 (50.2)	.002
History of MI, n (%)	2,351 (15.6)	2,036 (15.4)	315 (17.0)	.09	1,810 (16.2)	541 (13.9)	.001
History of PCI, n (%)	2,126 (14.2)	1,860 (14.1)	266 (14.4)	.76	1,655 (14.9)	471 (12.1)	< .001
History of CABG, n (%)	531 (3.5)	464 (3.5)	67 (3.6)	.83	425 (3.8)	106 (2.7)	.002
**Clinical presentation**							
STEMI	9,019 (59.8)	7,950 (60.2)	1,069 (57.5)	.03	6,874 (61.5)	2,145 (55.1)	< .001
Non-STEMI	6,054 (40.2)	5,265 (39.8)	789 (42.5)		4,305 (38.5)	1,749 (44.9)	
PCI, n (%)	14,168 (94.0)	12,488 (94.5)	1,680 (90.4)	< .001	10,870 (97.2)	3,298 (84.7)	< .001
CABG, n (%)	1,024 (6.8)	821 (6.2)	203 (10.9)	< .001	355 (3.2)	669 (17.2)	< .001
Primary PCI among STEMI, n (%)	7,577 (84.0)	6,688 (84.1)	889 (83.2)	.42	5,801 (84.4)	1,776 (82.8)	.08
Killip class I/II, n (%)	13,992 (92.8)	12,289 (93.0)	1,703 (91.7)	.04	10,449 (93.5)	3,543 (91.0)	< .001
Killip class III/IV, n (%)	1,079 (7.2)	924 (7.0)	155 (8.3)		729 (6.5)	350 (9.0)	
Serum creatinine in umol/L, median (IQR)	88 (75–105)	88 (75–105)	90 (76–108)	< .001	87 (74–104)	89 (76–109)	< .001
LVEF < 50%, n (%)	7,321 (54.2)	6,468 (54.6)	853 (51.0)	.005	5,413 (53.8)	1,908 (55.3)	.14
LVEF < 30%, n (%)^a^	1,045 (8.5)	902 (8.3)	143 (9.5)	.118	723 (7.9)	322 (10.3)	< .001
LVEF in %, median (IQR)^a^	45 (38–55)	50 (38–55)	45 (38–55)	.07	45 (35–55)	45 (40–55)	.005
**Medication given at discharge**							
Aspirin, n (%)	14,509 (96.3)	12,767 (96.6)	1,742 (93.8)	< .001	10,860 (97.2)	3,649 (93.7)	< .001
P2Y12 inhibitors, n (%)	14,433 (95.8)	12,685 (96.0)	1,748 (94.2)	< .001	10,943 (97.9)	3,490 (89.7)	< .001
Beta-blocker, n (%)	13,215 (87.7)	–	–	–	10,063 (90.0)	3,152 (81.0)	< .001
ACEI/ARB, n (%)	11,179 (74.2)	10,063 (76.2)	1,116 (60.1)	< .001	–	–	–
Lipid lowering drugs, n (%)	14,725 (97.7)	12,952 (98.0)	1,773 (95.4)	< .001	10,982 (98.2)	3,743 (96.1)	< .001

*ACEI* angiotensin converting enzyme inhibitor, *ARB* angiotensin receptor blocker, *CABG* coronary artery bypass graft, *HF* heart failure, *IQR* interquartile range, *LVEF* left ventricular ejection fraction, *MI* myocardial infarction, *PCI* percutaneous coronary intervention, *STEMI* ST-segment elevation myocardial infarction.

^a^Data was from 2008 to 2013.

At 12 months of follow up, MACE occurred in 1,671 (11.1%) patients. A total of 525 (3.5%) patients died, 774 (5.1%) patients were hospitalized for HF and 600 (4.0%) patients were hospitalized for MI. BB use at discharge was associated with a lower incidence of the combined MACE endpoint (adjusted HR 0.80, 95% CI 0.70–0.93) and all-cause mortality (adjusted HR 0.69, 95% CI 0.55–0.88) while ACEI/ARB use at discharge was associated with lower all-cause mortality (adjusted HR 0.80, 95% CI 0.66–0.98) and lower hospitalization for HF (adjusted HR 0.80, 95% CI 0.68–0.95). Neither BB nor ACEI/ARB at discharge was associated with lower incidence of hospitalization for MI (Table 2). We next compared MACE and the individual endpoints among the following four groups: BB + ACEI/ARB, BB only, ACEI/ARB only and no BB + no ACEI/ARB group (reference group). The BB + ACEI/ARB group had the lowest incidence of MACE (adjusted HR 0.70, 95% CI 0.57–0.86), all-cause mortality (adjusted HR 0.55, 95% CI 0.40–0.77) and hospitalization for HF (adjusted HR 0.64, 95% CI 0.48–0.86) among all four groups. BB only was associated with a lower incidence of MACE (adjusted HR 0.74, 95% CI 0.59–0.92) and all-cause mortality (adjusted HR 0.64, 95% CI 0.45–0.92) than the no ACEI/ARB + no BB group. ACEI/ARB-only was associated with a lower incidence of hospitalization for HF (adjusted HR 0.67, 95% CI 0.45 -0 0.99) than the no ACEI/ARB + no BB group. There was no difference in the incidence of MI in either of the first three treatment groups when compared with the no BB + no ACE/ABR group (Table 2).

**Table 2:** Crude and adjusted event rates among patients given BB and ACEI/ARB at discharge.

Adverse events	BB		ACEI/ARB		BB and ACEI/ARB			
	Yes (n = 13,215)	No (n = 1,858)	Yes (n = 11,179)	No (n = 3,894)	BB + ACEI/ARB (n = 10,063)	BB only (n = 3,152)	ACEI/ARB only (n = 1,116)	No BB + No ACEI/ARB (n = 742)
**MACE**								
Crude event rates, n (%)	1,418 (10.7)	253 (13.6)	1,206 (10.8)	465 (11.9)	1,065 (10.6)	353 (11.2)	141 (12.6)	112 (15.1)
Unadjusted HR (95% CI)	0.77 (0.68–0.89)	1.00 (ref)	0.89 (0.80–0.99)	1.00 (ref)	0.68 (0.56–0.82)	0.72 (0.59–0.90)	0.82 (0.64–1.05)	1.00 (ref)
Adjusted HR (95% CI)^a^	0.80 (0.70–0.93)	1.00 (ref)	0.91 (0.81–1.02)	1.00 (ref)	0.70 (0.57–0.86)	0.74 (0.59–0.92)	0.80 (0.62–1.05)	1.00 (ref)
**All-cause mortality**								
Crude event rates, n (%)	421 (3.2)	104 (5.6)	359 (3.2)	166 (4.3)	305 (3.0)	116 (3.7)	54 (4.8)	50 (6.7)
Unadjusted HR (95% CI)	0.57 (0.46–0.71)	1.00 (ref)	0.75 (0.62–0.90)	1.00 (ref)	0.44 (0.33–0.60)	0.54 (0.39–0.76)	0.71 (0.48–1.04)	1.00 (ref)
Adjusted HR (95% CI)^a^	0.69 (0.55–0.88)	1.00 (ref)	0.80 (0.66–0.98)	1.00 (ref)	0.55 (0.40–0.77)	0.64 (0.45–0.92)	0.72 (0.47–1.10)	1.00 (ref)
**HF hospitalization**								
Crude event rates, n (%)	666 (5.0)	108 (5.8)	540 (4.8)	234 (6.0)	488 (4.9)	178 (5.7)	52 (4.7)	56 (7.6)
Unadjusted HR (95% CI)	0.86 (0.70–1.05)	1.00 (ref)	0.80 (0.68–0.93)	1.00 (ref)	0.63 (0.48–0.83)	0.74 (0.55–1.00)	0.61 (0.42–0.89)	1.00 (ref)
Adjusted HR (95% CI)^a^	0.84 (0.68–1.03)	1.00 (ref)	0.80 (0.68–0.95)	1.00 (ref)	0.64 (0.48–0.86)	0.76 (0.55–1.04)	0.67 (0.45–0.99)	1.00 (ref)
**MI hospitalization**								
Crude event rates, n (%)	521 (3.9)	79 (4.3)	461 (4.1)	139 (3.6)	403 (4.0)	118 (3.7)	58 (5.2)	21 (2.8)
Unadjusted HR (95% CI)	0.92 (0.73–1.17)	1.00 (ref)	1.16 (0.96–1.40)	1.00 (ref)	1.42 (0.91–2.20)	1.32 (0.83–2.11)	1.85 (1.12–3.06)	1.00 (ref)
Adjusted HR (95% CI)^a^	0.98 (0.76–1.28)	1.00 (ref)	1.20 (0.97–1.49)	1.00 (ref)	1.44 (0.89–2.33)	1.27 (0.77–2.12)	1.71 (0.99–2.96)	1.00 (ref)

*ACEI* angiotensin converting enzyme inhibitor, *ARB* angiotensin receptor blocker, *CI* confidence interval, *HF* heart failure, *HR* hazard ratio, *MI* myocardial infarction.

^a^Adjusted for age, sex, race, history of diabetes, history of hypertension, history of hyperlipidemia, history of myocardial infarction/percutaneous coronary intervention/coronary artery bypass graft, smoking status, Killip class on presentation, serum creatinine on presentation, LVEF < 50% during hospitalization.

**Figure 2:**
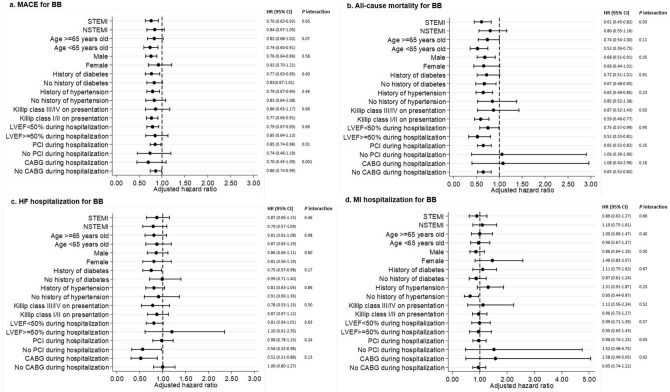
Adjusted risk of MACE among patients given BB at discharge compared to those not given BB at discharge (reference group) by subgroups. *ACEI/ARB* Angiotensin converting enzyme inhibitors/angiotensin receptor blockers, *AMI* acute myocardial infarction, *BB* beta-blockers, *CABG* coronary artery bypass graft, *CI* confidence interval, *HF* heart failure, *HR* hazard ratio, *LVEF* left ventricular ejection fraction, *MACE* major adverse cardiovascular events, *MI* myocardial infarction, *NSTEMI* non-ST-segment elevation myocardial infarction, *STEMI* ST-segment elevation myocardial infarction, *PCI* percutaneous coronary intervention. This figure was performed with STATA SE software, version 13.

**Figure 3:**
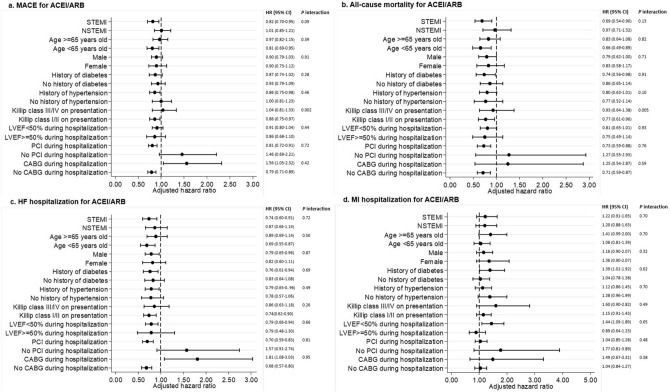
Adjusted risk of MACE among patients given ACEI/ARB at discharge compared to those not given ACEI/ARB at discharge (reference group) by subgroups. *ACEI/ARB* Angiotensin converting enzyme inhibitors/angiotensin receptor blockers, *AMI* acute myocardial infarction, *BB* beta-blockers, *CABG* coronary artery bypass graft, *CI* confidence interval, *HF* heart failure, *HR* hazard ratio, *LVEF* left ventricular ejection fraction, *MACE* major adverse cardiovascular events, *MI* myocardial infarction, *NSTEMI* non-ST-segment elevation myocardial infarction, *STEMI* ST-segment elevation myocardial infarction, *PCI* percutaneous coronary intervention. This figure was performed with STATA SE software, version 13.

The treatment effect for the composite MACE and individual endpoints of all-cause mortality, MI and HF hospitalization was largely consistent and homogenous across all subgroups for BB (Fig. 2) and ACEI/ARB (Fig. 3). Of note, the association of BB at discharge with lower mortality (Fig. 2b) and ACEI/ARB at discharge lower hospitalization for HF (Fig. 3c) was consistent across LVEF subgroups of < 50% and ≥ 50%. The subgroup analysis showed that the risk of mortality and MI hospitalization associated with BB prescribed at discharge were higher in the subgroup who underwent revascularization with CABG as compared to PCI. Conversely, HF hospitalization associated with BB was lower in patients who underwent CABG as compared to PCI. In patients who receive ACEI/ARB, the risk of mortality, HF and MI hospitalization were consistently higher in the subgroup who underwent revascularization with CABG as compared to PCI.

## Discussion

BB and ACEI/ARB may confer additive effects on survival in patients with AMI via different mechanisms: BB inhibits sympathetic activity and guards against arrhythmic deaths while ACEI/ARB modifies cardiac remodelling and reduces HF mortality^20^. In this contemporary cohort of patients with AMI undergoing inhospital revascularization, we observed that prescription of either BB or ACEI/ARB at discharge was associated with a lower incidence of 12-month all-cause mortality compared with patients who received neither drug at discharge, even after adjusting for relevant confounding variables. ACEI/ARB use at discharge, with or without BB, was associated with a reduced incidence of hospitalization for HF. Neither BB nor ACEI/ARB was associated with incidence of MI. The prescription of both BB and ACEI/ARB was associated with the lowest incidence of 12-month MACE compared with the prescription of BB only, ACEI/ARB only or neither BB nor ACEI/ARB at discharge. These findings were consistent across subgroups of reduced (< 50%) and preserved (≥ 50%) LVEF.

Pivotal trials of BB in AMI were largely conducted prior to routine invasive management of AMI^1^. The more contemporary CAPRICORN trial (Effect of Carvedilol on Outcome after Myocardial Infarction in Patients with Left Ventricular Dysfunction) demonstrated 23% reduction in mortality for post-MI patients with reduced LVEF^21^. In addition, several contemporary AMI registries reported lower mortality in those treated with BB, but only in those with reduced LVEF or evidence of HF^22,23^. Conversely, for preserved LVEF, data from the Myocardial Ischaemia National Audit Project, Reduction of Atherothrombosis for Continued Health and French Registry on Acute ST-segment elevation, Non-ST elevation myocardial infarction registries and post hoc analysis from the CHARISMA trial demonstrated no mortality benefit associated with the routine use of BB^8,9,11,24^. Compared to our study cohort, these studies had lower use of secondary prevention drugs among their subjects and included patients who did not undergo revascularization. Ongoing randomized controlled trials of routine BB use in patients with AMI and preserved LVEF, including the REDUCE-SWEDEHEART trial (NCT03278509) and the REBOOT-CNIC trial (NTC03596385), will hopefully address this knowledge gap.

Historically, randomized controlled trials of ACEI/ARB have demonstrated a reduction in mortality and hospitalization for HF among patients with AMI and both reduced LVEF^4^ as well as preserved LVEF^7,25^. In contrast, several contemporary observational studies have found no reduction in mortality or hospitalization for HF associated with ACEI/ARB among patients with AMI and preserved LVEF^26,27^. Several unique features of our study population should be noted when compared with these aforementioned observational studies: there was a high prevalence of cardiovascular risk factors (31.4% diabetes, 57.5% hypertension, and 52.4% hyperlipidemia), all study participants underwent inhospital revascularization, and we were able to compare clinical outcomes between patients prescribed combination treatment with BB + ACEI/ARB, BB only, ACEI/ARB only versus no BB + no ACEI/ARB. One other observational study that looked at AMI patients who underwent PCI in South Korea (N = 33,390) found no significant differences in the adjusted risk of all-cause mortality for patients treated with BB + ACEI/ARB, BB-only, ACEI/ARB-only as compared to non-treatment^28^. However, they only included patients who survived 30 days after the index procedure, which may result in low risk patients being selected. This study also did not examine other clinical outcomes such as hospitalization for HF or MI.

Several other findings in our study desire elaboration. First, we found that neither BB nor ACEI/ARB prescription at discharge was associated with a reduction in subsequent hospitalization for MI (Table 2). We postulate that only including revascularized AMI patients with high usage of dual antiplatelet therapy and lipid lowering drugs could have mitigated their risk of future MI, resulting in similar incidence of recurrent MI between patients who were and were not treated with BB or ACEI/ARB. Second, the subgroup analysis showed that the mortality benefit associated with BB (Fig. 2b) or ACEI/ARB (Fig. 3b) prescription at discharge was greater among patients in Killip class I/II at presentation compared with patients in Killip class III/IV at presentation (Interaction *p* 0.02 for BB and 0.005 for ACEI/ARB). Here, we hypothesize that a cautious approach is needed when prescribing either BB or ACEI/ARB to patients with severe acute heart failure or cardiogenic shock. It is unknown whether patients in the higher Killip classes from our study might have been more susceptible to systemic arterial hypotension from early BB or ACEI/ARB use, worse HF from BB use or acute kidney injury and/or hyperkalemia from ACEI/ARB use. We therefore recommend that early post-discharge monitoring of blood pressure, renal function and potassium be routine in this high risk group of patients. Third, BB and ACEI/ARB therapies were associated with better clinical outcomes despite the differences in baseline demographics between the group who were treated versus those who were not. Patients treated with BB had a lower risk profile (younger patients, fewer smokers) than those who were not treated with BB, whereas patients treated with ACEI/ARB had a higher risk profile (more diabetes, hypertension, hyperlipidemia, previous MI, previous PCI, and previous CABG) than those who were not treated with ACEI/ARB. These differences in baseline risk between treatment groups may have enhanced the benefit associated with BB use and diminished the benefit associated with ACEI/ARB use because of confounding. Forth, the subgroup analysis showed that in patients prescribed with BB or ACEI/ARB at discharge, risk of adverse clinical outcomes (except HF hospitalization in BB) was higher in the group who underwent revascularization with CABG as compared to PCI. It is likely that patients who underwent CABG were sicker at presentation due to multivessel coronary artery disease. They would also tend to have delayed intervention as compared to PCI resulting in loss of myocardium.

Our study represents a contemporary national registry with high standards of background care in pharmacotherapy. In addition, 24-h nationwide primary PCI has been the standard of care for STEMI in Singapore since 2007. To our knowledge, our cohort is the largest registry of AMI who underwent inhospital revascularization that study the association of BB and ACEI/ARB with MACE, allowing robust subgroup comparisons. Our study has several limitations. First, the nonrandomized nature of the study could have resulted in selection bias in treatment allocation. Because the decision to initiate BB and/or ACEI/ARB therapy were dependent on physicians’ preferences, institutional protocols, and patients’ tolerability of the drugs, conclusions on causality cannot be made. Second, there could also be certain unmeasured and unknown confounders that can influence the study outcomes including unrecorded co-morbidities, medication adherence and socioeconomic status. Multivariate analyses may have incompletely adjusted for these differences. Third, the registry captures whether contraindications excluded patients from receiving BB or ACEI/ARB but did not systematically capture the specific reasons for contraindications such as systemic hypotension, bradycardia, asthma, advance chronic kidney disease or hyperkalemia. Fourth, the results were based on the class effect of BB and/or ACEI/ARB. We do not have the data on the type of BB and/or ACEI/ARB that were initiated. Fifth, longitudinal data on up-titration of dosage, cessation of therapy and long-term adherence to BB and/or ACEI/ARB were not captured into the registry. Sixth, we only analysed a study cohort up to year 2013 so a more contemporary study population would have been preferable. Finally, the ethnic distribution of our study cohort may limit its generalization to other ethnic population, especially in the West.

## Conclusion

Among patients with AMI who are managed with inhospital revascularization and who are receiving high levels of dual antiplatelet and lipid-lowering therapy, either BB or ACEI/ARB use was associated with a lower incidence of 12-month all-cause mortality than if neither drug was prescribed at discharge. Use of ACEI/ARB, with or without BB, was associated with a reduced incidence of HF hospitalization. The greatest reduction in MACE was observed among patients receiving both BB and ACEI/ARB at discharge, regardless of LVEF. Our study findings conflict with that of other contemporary observation studies, highlighting continuing knowledge gaps in the appropriate use of BB and ACEI/ARB among patients with AMI in the current treatment era. While randomized trials of BB are ongoing in contemporarily-managed AMI populations, we propose that the role ACEI/ARB treatment also be re-examined in randomized trials of patients with AMI who undergo early revascularization and are already receiving high levels of dual antiplatelet and lipid-lowering therapy.
